# Inflammatory Status Assessment by Machine Learning Techniques to Predict Outcomes in Patients with Symptomatic Aortic Stenosis Treated by Transcatheter Aortic Valve Replacement

**DOI:** 10.3390/diagnostics13182907

**Published:** 2023-09-11

**Authors:** Alexandru Stan, Paul-Adrian Călburean, Reka-Katalin Drinkal, Marius Harpa, Ayman Elkahlout, Viorel Constantin Nicolae, Flavius Tomșa, Laszlo Hadadi, Klara Brînzaniuc, Horațiu Suciu, Marius Mărușteri

**Affiliations:** 1 Emergency Institute for Cardiovascular Diseases and Transplantation Târgu Mureş, 540136 Târgu Mureş, Romania; alexandru.stan@umfst.ro (A.S.); reka_kata@yahoo.com (R.-K.D.);; 2 University of Medicine, Pharmacy, Science and Technology “George Emil Palade” of Târgu Mureş, 540139 Târgu Mureş, Romania

**Keywords:** inflammatory markers, machine learning, transcatheter aortic valve replacement

## Abstract

(1) Background: Although transcatheter aortic valve replacement (TAVR) significantly improves long-term outcomes of symptomatic severe aortic stenosis (AS) patients, long-term mortality rates are still high. The aim of our study was to identify potential inflammatory biomarkers with predictive capacity for post-TAVR adverse events from a wide panel of routine biomarkers by employing ML techniques. (2) Methods: All patients diagnosed with symptomatic severe AS and treated by TAVR since January 2016 in a tertiary center were included in the present study. Three separate analyses were performed: (a) using only inflammatory biomarkers, (b) using inflammatory biomarkers, age, creatinine, and left ventricular ejection fraction (LVEF), and (c) using all collected parameters. (3) Results: A total of 338 patients were included in the study, of which 56 (16.5%) patients died during follow-up. Inflammatory biomarkers assessed using ML techniques have predictive value for adverse events post-TAVR with an AUC-ROC of 0.743 and an AUC-PR of 0.329; most important variables were CRP, WBC count and Neu/Lym ratio. When adding age, creatinine and LVEF to inflammatory panel, the ML performance increased to an AUC-ROC of 0.860 and an AUC-PR of 0.574; even though LVEF was the most important predictor, inflammatory parameters retained their value. When using the entire dataset (inflammatory parameters and complete patient characteristics), the ML performance was the highest with an AUC-ROC of 0.916 and an AUC-PR of 0.676; in this setting, the CRP and Neu/Lym ratio were also among the most important predictors of events. (4) Conclusions: ML models identified the CRP, Neu/Lym ratio, WBC count and fibrinogen as important variables for adverse events post-TAVR.

## 1. Introduction

Degenerative aortic valve stenosis (AS) is the most commonly acquired valvular heart disease and its prevalence increases with an ageing population [1]. Once AS becomes symptomatic, a poor prognosis is observed, with a survival rate of 30% at 5 years [2]. The only treatment option for decades was surgical aortic valve replacement (SAVR) with good long-term prognosis in ideal candidates. However, the operative risk is heterogenous, significantly increasing with old age and association of cardiac or non-cardiac comorbidities [3], leading to a deferral from SAVR in a third of the patients with symptomatic AS [4]. Transcatheter aortic valve replacement (TAVR) procedure, which was only relatively recently introduced in clinical practice, is nowadays generally accepted as the new standard of care for patients with symptomatic severe AS who are not candidates for open surgery [5]. Although TAVR significantly improves the long-term outcomes of symptomatic severe AS patients, reported 3-years mortality rates are roughly 40% [6,7]. Thus, identifying predictors of adverse events post-TAVR, especially modifiable parameters, is a major clinical desiderate.

Severe AS diagnosis is performed using transthoracic echocardiographic evaluation of the mean aortic transvalvular gradient, peak aortic transvalvular velocity, and aortic valve area [8]. In certain clinical conditions, echocardiography is not enough, and cardiac computed tomography contributes to the final diagnosis. Severe AS is diagnosed in the following three clinical presentations [8]: (1) High-gradient AS—mean aortic transvalvular gradient above 40 mmHg, peak aortic transvalvular velocity above 4.0 m/s, and aortic valve area less than 1 cm^2^. All high-gradient AS cases are considered severe AS, irrespective of left ventricular ejection fraction (LVEF) or LV flow conditions. (2) Low-flow, low-gradient AS, reduced LVEF—mean aortic transvalvular gradient below 40 mmHg, aortic valve area less than 1 cm^2^, a LVEF below 50% and an indexed stroke volume less than 35 mL/m^2^. This clinical instance requires further investigation to determine whether the low aortic valve area is due to low-flow conditions and a dobutamine test should be performed. If under dobutamine, the aortic valve area remains under 1 cm^2^ with a minimum of 20% increase in stroke volume, severe AS can be considered. (3) Low-flow, low-gradient AS, preserved LVEF—mean aortic transvalvular gradient below 40 mmHg, aortic valve area less than 1 cm^2^, a LVEF above 50% and an indexed stroke volume less than 35 mL/m^2^. The definite diagnosis of severe AS is relatively more difficult and prognosis of this clinical form of AS is similar to high-gradient AS [9], although this clinical instance is less frequent. High degrees of aortic valve calcifications at cardiac computed tomography provide important further diagnostic elements [8].

Machine learning (ML) techniques were described decades ago [10], but only recently gained exponential attention because of the increase in computational power and the availability of big data [11]. Machine learning techniques include, but are not limited to, algorithms such as random forest, gradient boosting machines or support vector machines [12]. Machine learning models differ from classical statistical methods such as logistical regression by their capacity to make predictions on unseen data [12]. Machine learning models can be used to perform either classification (binary or multiclass predictions) or regression (predicting a value). The use of ML techniques is appealing because it can effectively handle non-linearity and find complex interaction patterns among numerous variables, thus offering the potential to improve prediction accuracy [12,13]. However, due to its underlying mathematical complexity, ML models are difficult to interpret, being considered a black box [14]. In cardiovascular medicine, ML models can identify complex interactions among clinical variables and make an accurate event prediction [15]. The aim of our study was to identify potential inflammatory biomarkers with predictive capacity for post-TAVR event prediction from a wide panel of routine biomarkers by employing ML techniques.

## 2. Materials and Methods

All patients diagnosed with symptomatic severe AS and treated by TAVR since January 2016 at the Emergency Institute for Cardiovascular Diseases and Transplantation of Târgu Mureş were included in the present study. Patient data was retrospectively collected and included baseline demographic characteristics, cardiovascular risk factors, comorbidities, laboratory parameters on admission, echocardiographic parameters, coronary anatomy parameters, TAVR-related parameters, and clinical post-procedural evolution. A total of 93 clinical parameters were included in the ML analysis. Patients were not eligible for TAVR procedure if certain criteria were present, such as active infection, severe comorbidities, a high grade of frailty, severely reduced cognitive function, or limited life expectancy, consistent with our institutional TAVR protocol. The Romanian National Health Insurance System database supplied mortality rates for all the patients. For patients who had died during follow-up, the Regional Statistics Office of the Romanian National Institute of Statistics supplied the exact date and cause of death according to the tenth revision of the International Classification of Diseases (ICD-10). All included patients completed informed consent forms. The study was approved by the ethical committee of our institution. The protocol was carried out in accordance with the ethical principles for medical research involving human subjects established by the Declaration of Helsinki, protecting the confidentiality of personal information of the patients.

### 2.1. Machine Learning

A gradient boosting algorithm (XGBoost) was used to train (1) a model as a binary classifier for predicting 3-year all-cause cause mortality and (2) an accelerated failure time (AFT) model to predict survival. Open-source XGBoost native package was implemented in Python version 3.9. The model was trained using a 5-fold cross-validation technique. Predictions from the testing dataset for all 5 folds were pooled when performance was assessed. Hyperparameter optimization was obtained using grid search technique. No conversion of any data to a specific format was performed and one-hot encoding was used when dealing with categorical variables. Prediction interpretation and visualization was performed using open-source Shapley additive explanations (SHAP) framework that was also implemented in Python version 3.9. Three separate analyses were performed: (1) an analysis using only inflammatory biomarkers, (2) an analysis using inflammatory biomarkers, age, creatinine, and left ventricular ejection fraction (LVEF), and (3) an analysis using all collected parameters.

### 2.2. Statistical Analysis

A significance level α of 0.05 and a 95% confidence interval (CI) were considered. Continuous variables were evaluated for normal distribution using the Shapiro-Wilk test. Continuous variables with parametric distributions were reported as mean ± standard deviation and compared using a non-paired or paired Student *t*-test, while continuous variables with non-parametric distributions and discrete variables were reported as the median (interquartile range) and compared using a Mann–Whitney or Wilcoxon test. Categorical variables were reported as absolute and relative frequencies and compared using Fisher exact test for variables with frequencies of less than 5, and a Chi^2^ test otherwise. The prediction performance of ML models were evaluated using multiple performance metrics: area under the receiver–operator characteristic (AUC-ROC), area under the precision–recall curve (AUC-PR). Statistical analysis was performed using R version 4.1.1 and R Studio version 1.4.17.

## 3. Results

**Figure 1:**
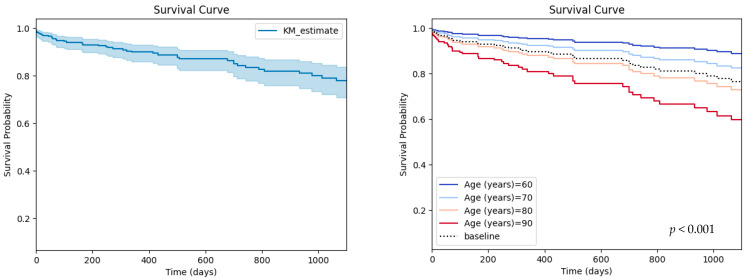
Survival curve for the studied population (**left**) and partial effects of age on survival (**right**).

A total number of 338 patients were included, of which 204 (60.3%) were males, with a median age of 76 (72–80) years and median body mass index of 29.01 ± 4.48 kg/m^2^. The baseline characteristics of the studied population are reported in Table 1 and the survival curve is illustrated in Figure 1.

During follow-up, a total of 56 (16.5%) patients died, of which 3 (0.8%) patients suffered in-hospital death during their initial hospitalization for the TAVR procedure. There was no patient–prosthesis mismatch in the studied population.

Echocardiographic parameters are reported in Table 2. Among significant echocardiographic parameters besides LVEF, left ventricular end-diastolic diameter (LVEDD) was also higher among patients who died during follow-up, while baseline aortic gradients were not predictive of death.

Cardiac computed tomography parameters relevant for the TAVR population are reported in Table 3. Interestingly, none of the baseline LVOT or aortic root parameters were predictive of adverse events, while a higher calcium score of the left main coronary artery was predictive of impaired survival. 

A wide range of routinely performed laboratory parameters were determined. Of those, inflammatory related parameters (Table 4) had a predictive value for clinical evolution after TAVR.

Biochemical parameters with potentially important survival effects are reported in Table 5. Serum creatinine and serum albumin were significantly higher and lower, respectively, in patients who suffered all-cause death during follow-up.

### Machine Learning Assessment

**Figure 2:**
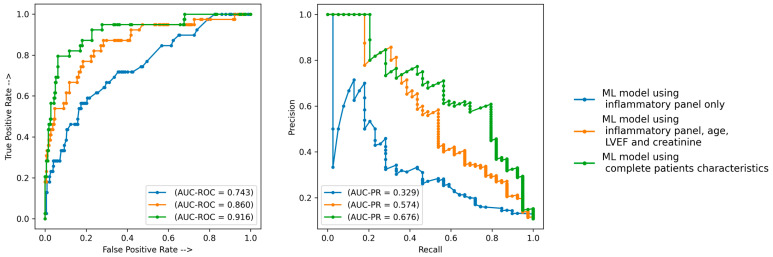
Prediction performance of the ML models. AUC-PR—area under precision recall curve; AUC-ROC—area under receiver operator curve; ML—machine learning.

Inflammatory biomarkers assessed using ML techniques had a predictive value for adverse events post-TAVR, with an AUC-ROC of 0.743 and an AUC-PR of 0.329 (Figure 2). When adding age, creatinine and LVEF to the inflammatory panel, the ML performance increased to an AUC-ROC of 0.860 and an AUC-PR of 0.574 (Figure 2). If using the entire dataset (inflammatory parameters and complete patient characteristics), the ML performance was the highest with an AUC-ROC of 0.916 and an AUC-PR of 0.676 (Figure 2). 

**Figure 3:**
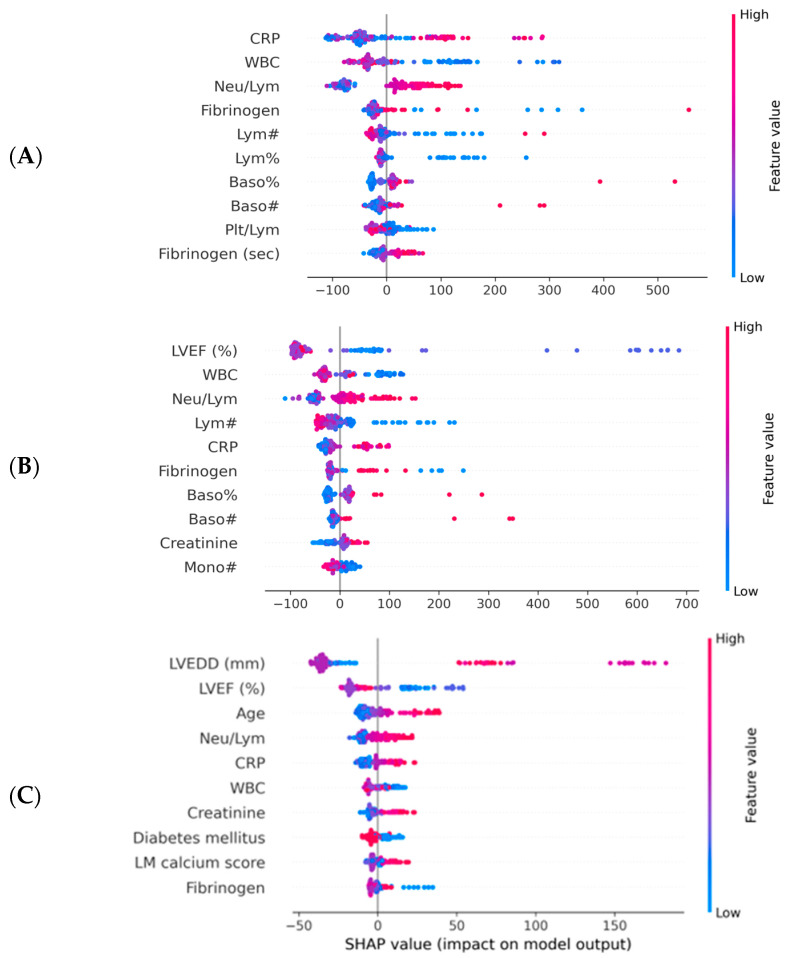
Importance plots for the ML models. CRP—C reactive protein; LM—left main artery; LVEF—left ventricular ejection fraction; LVEDD—left ventricular end diastolic diameter; ML—machine learning; WBC—white blood cells. (**A**) Importance plot for ML model applied to dataset with inflammatory markers. (**B**) Importance plot for ML model applied to dataset with inflammatory markers, LFEV, age and creatinine. (**C**) Importance plot for ML model applied to entire dataset.

**Figure 4:**
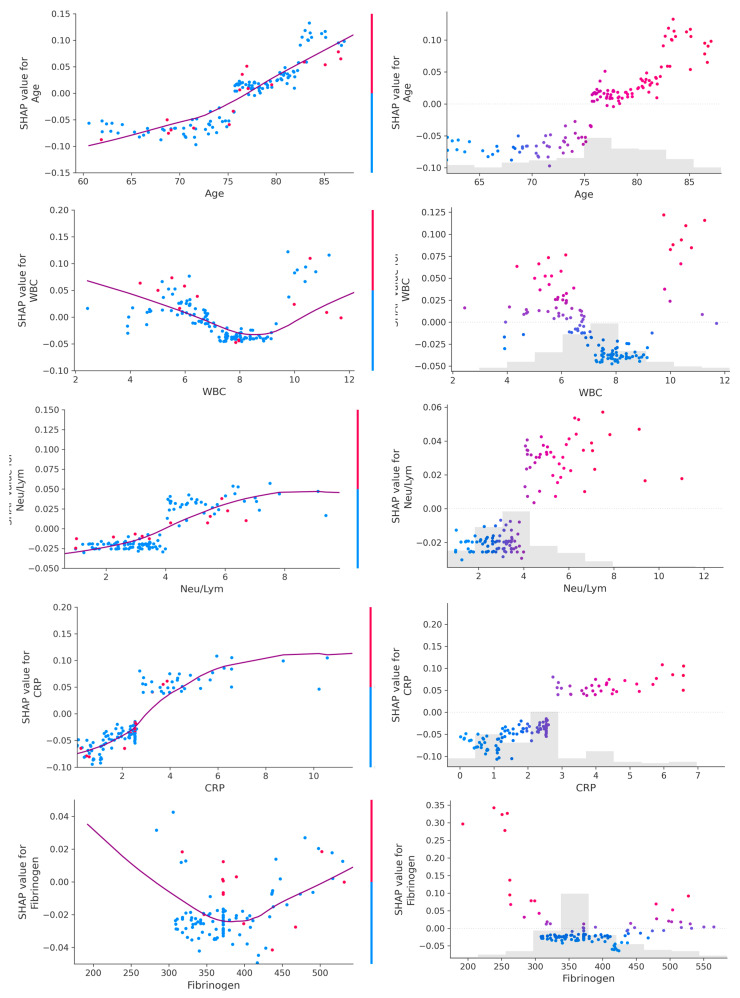
Dependance plot (**left**) and scatter plot (**right**) for age and inflammatory parameters. A linear relationship can be observed between values of age, CRP and Neu/Lym ratio and their SHAP values, while a bimodal relationship can be observed between values of WBC and fibrinogen and their SHAP values.

Of note, the tuned hyperparameters of the final models included (1) a total of 300 decision trees aggregated, (2) a tree depth of four levels and (3) a learning rate of 0.01. The ML decision process can be understood using Shapley values [16]. Initially, the ML model ranks the most important variables for the prediction of mortality (Figure 3). On the dataset with only inflammatory markers, C-reactive protein (CRP), white blood cells (WBC) count and Neu/Lym ratio were the three most important features (Figure 3A). On the dataset with inflammatory markers, age, creatinine and left ventricular ejection fraction (LVEF), LVEF, CRP and WBC were the three most important features (Figure 3B). On the dataset with complete patient characteristics, left ventricular end-diastolic diameter (LVEDD), LVEF, CRP and Neu/Lym ratio were the most important features (Figure 3C). Afterwards, each variable was assigned a SHAP value for a particular variable value. A lower SHAP value is protective, while a higher score reflects impaired prognosis. In Figure 3, the x-axis reflects SHAP values, while each parameter has a blue and red side reflecting lower and higher parameter values, respectively. For instance, the blue side of the LVEDD parameter reflects lower LVEDD values and is on negative side of SHAP values; thus, there is a better prognosis when the LVEDD is lower. In contrast, the blue side of the LVEF parameter reflects higher LVEF values and is on the positive side of SHAP values; thus, there is a worse prognosis when the LVEF is lower. The dependence plots between predictor value and SHAP value for the most important variables are illustrated in Figure 4.

## 4. Discussion

The main findings of our study can be summarized as follows: (1) inflammatory biomarkers assessed using ML techniques have predictive value for adverse events post-TAVR, with an AUC-ROC of 0.743 and an AUC-PR of 0.329; the most important variables were CRP, WBC count and the Neu/Lym ratio. (2) When adding age, creatinine and LVEF to an inflammatory panel, the ML performance increased to an AUC-ROC of 0.860 and an AUC-PR of 0.574; even though the LVEF was the most important predictor, inflammatory parameters retained their value. (3) When using the entire dataset (inflammatory parameters and complete patient characteristics), the ML performance was the highest, with an AUC-ROC of 0.916 and an AUC-PR of 0.676; in this setting, the CRP and Neu/Lym ratio were also among the most important predictors of events. (4) When using SHAP values to explain the outcomes, with the increase in age, CRP and Neu/Lym ratio, an increase in event risk was also observed, while for WBC count and fibrinogen levels, a bimodal relationship was observed—the event risk was higher for both low and high levels of both WBC and fibrinogen. Even though numerous echocardiographic and cardiac computed tomography parameters were also included in the final analysis, only the LVEF and LVEDD were among the most important predictors. Besides inflammatory markers, it is not surprising that age, echocardiographic parameters, and creatinine are other important clinical parameters, as it is commonly known that they are the main survival determinants of heart disease populations. Our study supports the concept of precision phenotyping—AI and ML techniques can find complex patterns and interactions among clinical parameters that are invisible or unimportant to the clinician. The performance analysis of the ML models showed that an accurate prognosis estimate was given from routine biomarkers. The performance in survival prediction was reflected not only by the AUC-ROC, but also by the AUC-PR, a better metric for imbalanced datasets (e.g., deceased patients were fewer than alive patients) [17,18,19].

Undisputedly, TAVR offers both short-term and long-term advantages over SAVR, especially in high-risk patients, but post-TAVR evolution does not lack adverse events. Growing evidence suggests that inflammation status both before and after TAVR is an important predictor of adverse outcomes. High levels of biomarkers such as CRP, GDF-15 or IL-8 were associated with a 1-year mortality after TAVR [20]. Similarly, impaired platelet activity after TAVR was also a predictor of mortality [21]. In our study, the CRP and Neu/Lym ratio were higher in patients who died during follow-up. Moreover, by employing ML models, a bimodal relationship was observed for WBC count—lower and higher values were associated with impaired survival (Table 3). This relationship was not observed when alive versus deceased patients were compared (Table 2). All included patients in the present study underwent transfemoral TAVR approach. A recent study reported that the inflammatory response was significantly lower in transfemoral TAVR compared to both SAVR and apical TAVR [22]. This reduced response in the context of transfemoral TAVR may also be responsible for the improved evolution of patients treated with this strategy. Noteworthy, patients were not subjected to TAVR procedure if there was an active infection or inflammation as per our institutional protocol and clinical guidelines. Our findings suggest that even subclinical inflammation assessed by routine biomarkers has important prognostic value. 

Inflammation is an important element in atherosclerotic disease that leads to aortic valve degeneration, thus being a potential and attractive pharmacologic target. In current clinical practice, pharmacological treatment in the context of symptomatic AS is directed to treating comorbidities since no pharmacological agent improves the clinical course of AS per se. The same principle is applied post-TAVR, with the exception of empirical double antiplatelet therapy for 3–6 months followed by indefinite single platelet inhibitor treatment [23]. Our observations, along with other evidence from literature, could sustain the hypothesis of a beneficial effect exerted by anti-inflammatory medication. Indeed, certain anti-inflammatory agents reduced the risk of cardiovascular events, such as colchicine, in the context of coronary artery disease [24]. 

The 3-year all-cause mortality or stroke rate was roughly 9% lower in TAVR versus SAVR high-risk patients [25]. However, TAVR mortality during follow-up was still high, at 32.9% in the same study [25]. In our study, mortality during follow-up was lower; however, the included population was also smaller. Nevertheless, the ML model, using all the clinical characteristics, predicted mortality with an AUC-ROC of 0.916. Low LVEF and high LVEDD were the two most important predictors, followed by a CRP and Neu/Lym ratio. A large meta-analysis also showed the impaired prognosis associated with low LVEF [26]. This is not surprising, since LVEF is the most important parameter of cardiac systolic function.

Our study is limited by the relatively small size of the study population, using all-cause instead of cardiovascular-cause mortality, and a lack of frailty scores. Including more patients would increase the statistical power of the study. However, some clear trends were observed for the studied parameters (Table 3).

## 5. Conclusions

Identifying predictors, especially modifiable ones, for impaired survival after TAVR for symptomatic severe AS is an important objective in contemporary cardiovascular medicine, since post-TAVR mortality is still relatively high. Inflammatory status assessment could provide such predictors. In our study, the ML models identified the CRP, Neu/Lym ratio, WBC count and fibrinogen as important variables for adverse events.

## Figures and Tables

**Table 1:** Baseline characteristics of the studied population.

Parameter	Entire Population (*n* = 338)	Alive at 3 Years (*n* = 282)	Deceased at 3 Years (*n* = 56)	*p*
Age (years)	76 (71–80)	76 (71–80)	78 ± 6	0.01
BMI (kg/m^2^)	29.01 ± 4.48	29.28 ± 4.52	26.02 ± 2.65	0.08
Male sex	204 (60.3%)	171 (60.6%)	33 (58.9%)	0.88
LVEF (%)	50 (40–60)	50 (40–60)	40 (35–55)	0.001
DCM	33 (9.76%)	22 (7.8%)	11 (19.64%)	0.01
CAD	202 (59.76%)	165 (58.51%)	37 (66.07%)	0.37
Previous MI	21 (6.21%)	16 (5.67%)	5 (8.93%)	0.36
Previous PCI	183 (54.1%)	151 (53.5%)	32 (57.1%)	0.66
Previous CABG	12 (3.55%)	10 (3.55%)	2 (3.57%)	0.99
Hypertension	270 (79.88%)	229 (81.21%)	41 (73.21%)	0.20
Diabetes mellitus	104 (30.77%)	79 (28.01%)	25 (44.64%)	0.01
Atrial fibrillation	99 (29.29%)	79 (28.01%)	20 (35.71%)	0.26
Stroke	19 (5.62%)	15 (5.32%)	4 (7.14%)	0.26
COPD	22 (6.51%)	19 (6.74%)	3 (5.36%)	0.99

BMI—body mass index; CABG—coronary artery bypass graft; CAD—coronary artery disease; COPD—chronic obstructive pulmonary disease; DCM—dilated cardiomyopathy; LVEF—left ventricular ejection fraction; MI—myocardial infarction; PCI—percutaneous coronary intervention.

**Table 2:** Comparison between pre-procedural echocardiographic parameters among studied groups.

Parameter	Entire Population (*n* = 338)	Alive at 3 Years (*n* = 282)	Deceased at 3 Years (*n* = 56)	*p*
LVEDD (mm)	50 (45–55.75)	50 (45–55)	54.05 ± 7.41	0.03
RVEDD (mm)	28 (24.75–32)	28 (24–31.25)	28.98 ± 6.22	0.45
PWT (mm)	13 (11–14)	13 (11–13.25)	12 (11.75–14)	0.23
IVST (mm)	14 (12–16)	14 (12–16)	14 (12–15)	0.65
Aortic annulus (mm)	21 (19–22)	21 (19–22)	21 (20–22)	0.49
Ascending aorta (mm)	33 (27–36)	33 (21–36)	33 (30.75–36.25)	0.98
LVOT diameter (mm)	20 (18–21)	20 (18–21)	20.65 ± 1.93	0.07
LA diameter (mm)	43 (37.75–47)	43 (36.75–47)	45 (39–49.25)	0.06
RA diameter (mm)	20 (15–30.75)	20 (15–29)	27.6 ± 16.31	0.23
Maximum gradient (mmHg)	71 (52.5–81)	72 (54.5–81.5)	66 (43.25–75.25)	0.42
PHT (ms)	300 (50–461)	270 (51.5–459)	335 (48.25–472.5)	0.44

LA—left atrium; LVEDD—left ventricular end-diastolic diameter; LVOT—left ventricular outflow tract; IVST—interventricular septum thickness; PHT—aortic pressure half-time; PWT—posterior wall thickness; RA—right atrium; RVEDD—right ventricular end-diastolic diameter.

**Table 3:** Comparison between cardiac computed tomography parameters among studied groups.

Parameter	Entire Population (*n* = 338)	Alive at 3 Years (*n* = 282)	Deceased at 3 Years (*n* = 56)	*p*
Annulus area (mm^2^)	510 (448.5–577.5)	509 (439–573)	573.67 ± 88.64	0.63
Annulus perimeter (mm)	82.36 ± 9.14	82 ± 9.27	86.37 ± 6.83	0.15
LVOT perimeter (mm)	81.2 (73.8–88.9)	80.6 (73.8–88.5)	85.23 ± 5.61	0.95
LVOT area (mm)	493 (414.5–591.25)	486.5 (405.75–585.75)	551.5 ± 69.39	0.29
Sinotubular diameter (mm)	28.79 ± 3.5	28.1 (26.9–30.7)	29.92 ± 4.57	0.78
LCA height (mm)	13.6 (12.4–16)	13.6 (12.7–16)	13.45 ± 2.89	0.81
RCA height (mm)	15.5 (13.25–18.4)	15.5 (13.4–18.2)	16.95 ± 4.55	0.44
LM calcium score	0 (0–111)	55 (0–72)	161 ± 61	0.02
LAD calcium score	246 (93–655)	239 (106–654)	403 ± 324	0.80
CX calcium score	13 (0–254)	10 (0–271)	136 ± 116	0.19
RCA calcium score	157 (24–444)	155 (21–441)	123 ± 44	0.17
Total coronary calcium score	689 (212–1336)	644 (213–1281)	983 ± 748	0.13

CX—circumflex artery; LCA—left coronary artery; LM—left main artery; LVOT—left ventricular outflow tract; RCA—right coronary artery.

**Table 4:** Comparison between inflammatory markers among studied groups.

Parameter	Entire Population (*n* = 338)	Alive at 3 Years (*n* = 282)	Deceased at 3 Years (*n* = 56)	*p*
WBC count (×10^3^/µL)	6.86 (5.94–8.36)	6.91 (6.06–8.35)	6.33 (5.44–8.46)	0.22
Neu (×10^3^/µL)	4.91 (4.08–5.96)	4.91 (4.11–5.95)	4.87 (3.9–6.53)	0.89
Lym (×10^3^/µL)	1.43 (1.14–1.87)	1.45 (1.15–1.89)	1.24 (0.81–1.59)	0.006
Neu (%)	66.3 (61.17–71.03)	66.28 (61–70.8)	68.03 (63.56–72.84)	0.98
Lym (%)	20 (16.3–24.01)	20.14 (16.42–24.17)	17.84 (12.75–22.03)	0.03
Neu/Lym ratio	3.43 (2.61–4.51)	3.41 (2.57–4.46)	3.99 (2.99–5.88)	0.04
Plt/Lym ratio	115.1 (91.4–150.1)	113.9 (90.8–147.6)	140.3 ± 66.4	0.12
CRP (mg/dL)	0.59 (0.16–1.39)	0.55 (0.15–1.33)	1.18 (0.52–2.39)	0.006
Fibrinogen (mg/dL)	371.9 (322.5–432.7)	371.9 (322.5–427.9)	392.6 ± 104.5	0.20
ESR (s)	27.7 (15–45)	28.3 (15–45)	28.0 ± 23.1	0.97

CRP—C-reactive protein; ESR—erythrocyte sedimentation rate; WBC—white blood cells.

**Table 5:** Comparison between biochemical parameters among studied groups.

Parameter	Entire Population (*n* = 338)	Alive at 3 Years (*n* = 282)	Deceased at 3 Years (*n* = 56)	*p*
Creatinine (mg/dL)	1.05 (0.88–1.31)	1.04 (0.88–1.29)	1.27 (0.98–1.53)	0.001
Total serum proteins (mg/dL)	6.6 (6.16–6.95)	6.57 ± 0.6	6.22 (5.98–6.91)	0.06
Serum albumin (mg/dL)	3.89 ± 0.44	3.93 ± 0.4	3.55 ± 0.54	0.001
Total serum CK (U/L)	80.67 (55.38–124.06)	81.5 (57.5–122.5)	78.4 (49.33–140)	0.73
Serum CK-MB (U/L)	19.42 (15.31–24.38)	19.42 (16–24)	19.25 (12.38–30.38)	0.81
Total bilirubin (mg/dL)	0.68 (0.51–0.88)	0.67 (0.51–0.87)	0.81 (0.56–1.23)	0.06
Cholesterol (mg/dL)	147 (125–180.75)	149.8 (127–181.12)	145.77 ± 41.66	0.10
LDL-cholesterol (mg/dL)	88.75 (72–115.25)	89.75 (72–115.62)	89.21 ± 30.57	0.35
HDL-cholesterol (mg/dL)	36.5 (30.83–44)	36.65 (31–44)	37.19 ± 12.14	0.43
Triglyceride (mg/dL)	98 (74.5–130.5)	99 (74.5–131)	92 (77.35–121.25)	0.26

CK-MB—Creatine kinase; CK-MB—Creatine kinase–MB isoform; HDL—high-density lipoprotein; LDL—low-density lipoprotein.

## Data Availability

Data available on request. The data and scripts underlying this article will be shared upon reasonable request by the corresponding author.
